# Across and Within- Individual Associations Between Everyday Activities and Quality of Life Relevant Measures, in Workers with Type 1 Diabetes

**DOI:** 10.1007/s11482-023-10171-2

**Published:** 2023-04-20

**Authors:** Raymond Hernandez, Stefan Schneider, Loree Pham, Elizabeth A. Pyatak

**Affiliations:** 1 Dornsife Center for Economic & Social Research, University of Southern California, 635 Downey Way, VPD 405, Los Angeles, CA 90089-3332 USA; 2 Department of Psychology, University of Southern California, Los Angeles, CA 90089 USA; 3 Chan Division of Occupational Science and Occupational Therapy, University of Southern California, Los Angeles, CA 90089 USA

**Keywords:** Ecological momentary assessment, Health promotion, Human activities, Type 1 diabetes, Workers

## Abstract

Relationships between activity engagement and health related quality of life (HRQOL) can differ based on the level of analyses. For instance, greater exercise on average may be linked with lower fatigue across individuals (between-person level), whereas the momentary experience of exercise may be associated with increased fatigue within an individual (within-person level). Disentangling the between- and within-person associations between everyday activities and HRQOL outcomes may provide insights for personalized lifestyle-oriented health promotion efforts for individuals with chronic conditions. The purpose of this paper was to examine the between- and within-person relationships between activity engagement and HRQOL relevant measures in a sample of 92 workers with type 1 diabetes (T1D), from whom we collected ecological momentary assessment (EMA) data 5–6 times daily over 14 days. At each EMA prompt, information was collected on the activity participants just engaged in, and HRQOL relevant metrics (e.g. mental health, blood glucose, fatigue, functioning). Momentary reports of “caring for others”, and more frequently “caring for others”, were both associated with decreased HRQOL. Reporting napping 10% or more of the time during a person’s waking hours, but not the momentary experience of napping, was associated with decreased HRQOL. Momentary reports of sleeping were associated with low activity satisfaction relative to other activities, but higher activity importance. Study results provided a quantitative representation of the lived experience of T1D covering multiple types of activity engagement, which potentially has health promotion implications for workers with T1D.

**Supplementary Information:**

The online version contains supplementary material available at 10.1007/s11482-023-10171-2.

## Introduction

Health related quality of life (HRQOL) is an important outcome considered by various institutions including the World Health Organization (World Health Organization, 2007) and the Centers for Disease Control and Prevention in the United States (Centers for Disease Control and Prevention, 2021). Various definitions and models of HRQOL exist (Bakas et al., 2012), but here we use its conceptualization as “how well a person functions in their life and his or her perceived wellbeing in physical, mental, and social domains of health” (Hays & Reeve, 2008, p.195). According to Ferrans and colleagues’ model, a multitude of factors can contribute to HRQOL (e.g., functional status, symptoms, and biological functioning), all of which are impacted by characteristics of both individuals and the environment (Ferrans et al., 2005). Consistent with the theorized multifaceted nature of HRQOL, the widely used Short Form Health Survey (SF-36) assesses HRQOL along eight dimensions including physical functioning, role limitations caused by physical health problems, role limitations caused by emotional problems, social functioning, emotional well-being, energy/fatigue, pain, and general health perceptions (Hays & Morales, 2001).

Daily activity engagement of various forms influences many HRQOL dimensions. For instance, more frequent physical activity has often been associated with greater HRQOL (Qi et al., 2020; Snedden et al., 2019). Higher engagement in social and leisure activities was linked to greater HRQOL in older adults (Horowitz & Vanner, 2010) and adults with HIV (Wion et al., 2022). More frequent participation in meaningful activities was linked with higher HRQOL in adults with mental health disorders (Goldberg et al., 2002). Note that all these studies investigated the relationship between people’s average activity engagement across time and HRQOL.

While the relationship between HRQOL and activity engagement between individuals has often been examined, the momentary relationships between HRQOL and activity engagement *within* individuals have not often been investigated. One study on workers found that, at the momentary level, the amount of happiness experienced during non-work activities played an important role in the degree to which they promoted recovery from work (Oerlemans & Bakker, 2014). Using ecological momentary assessment (EMA), repeated sampling of behaviors and experiences in real time in natural environments (Shiffman et al., 2008), Dunton et al. (2015) found that how momentary levels of positive affect were associated with physical activity depended on the immediate social and physical context. In another EMA study, higher negative affect and lower positive affect was associated with decreased physical activity at the momentary level (Smith et al., 2020).

Investigation of the associations between engagement in daily activities and HRQOL, at both the between-person and within-person levels, may help provide a more comprehensive picture of their relationship. Constructs can have different relationships depending on whether analyses address the association of their averages over time (i.e. between-person level), or their associations within a person (Curran & Bauer, 2011). For example, within a person, engagement in exercise may be associated with fatigue from the exertion, but greater overall engagement in exercise has been associated with decreased fatigue for a variety of populations with chronic conditions (Canário et al., 2016; Katz et al., 2018; Webel et al., 2016).

The greater clarity afforded by examining both within and between-person relationships between activity engagement and other HRQOL relevant variables can potentially yield insights for HRQOL interventions. For instance, consider the activity of napping. It has been associated with improvements in mood and decreased fatigue (Milner & Cote, 2009). However, napping has also been associated with increased risk for mortality (Leng et al., 2014). Examining within and between-person associations between napping and HRQOL relevant metrics would allow for evaluating whether these seemingly contradictory results are in part a result of the level of analyses. If napping was linked with mortality primarily because people with more severe health issues nap more frequently (between-person relationship), one implication may be that among relatively healthy populations, napping can be recommended to improve momentary mood and decrease momentary fatigue (within-person relationship), with less fear that this approach would result in negative long- term consequences.

Investigations of between- and within-person relationships between activity engagement and HRQOL may be especially useful in populations with chronic conditions. Over half (51.8%) of adults in the United States have at least one chronic condition (Boersma et al., 2020), with health promotion often recommended as a means to reduce the large economic and quality of life costs associated with chronic conditions (Levine, 2019). An improved understanding of the relationships between activity engagement and HRQOL in populations with chronic conditions may yield insights regarding potential targets for lifestyle interventions, a key aspect of health promotion (O’Donnell, 2009).

Adults with type 1 diabetes (T1D) represent one population that may benefit from investigations of the between- and within-person and relationships between activity engagement and HRQOL. T1D is characterized by destruction of beta cells in the pancreas, leading to absolute insulin deficiency (Kerner & Brückel, 2014). Individuals with T1D take on several tasks to manage their condition including checking blood glucose, engaging in regular physical activity, planning timing and content of meals, and regular dosing of insulin (Beck et al., 2017). Both health promotion efforts for T1D, and investigations of the lived experience of T1D, often focus on these specific self-management behaviors (Hansen et al., 2018; Hood et al., 2010; King et al., 2017; Quirk et al., 2014). However, from a Lifestyle Redesign® (LR) perspective (Pyatak et al., 2022), all daily activities can be impacted by T1D, and in turn affect overall health and well-being.

Examination of the between- and within-person relationships across a wide spectrum of activity engagements and HRQOL in adults with T1D may be particularly relevant for LR, an intervention framework that “promotes awareness of the relationship between everyday activities and health and guides people in the process of orchestrating occupations, habits, and routines to enhance health and well-being (Pyatak et al., 2022, p.2).” Interventions based on LR have been shown to improve the health and quality of life of various populations including adults with diabetes, chronic pain, and multiple sclerosis (Pyatak et al., 2022). Knowledge of the relationships between daily activity engagements and HRQOL could be particularly beneficial for this intervention framework, given its focus on the orchestration of daily activities for optimal well-being.

### Present Study

The purpose of this study was to examine the between- and within-person relationships between activity engagement and HRQOL relevant variables, in adult workers with type 1 diabetes (T1D), to provide a quantitative representation of the lived experience of T1D. In addition to the health management responsibilities associated T1D, workers with T1D must additionally cope with job demands, which can result in greater difficulty with diabetes management (Hansen et al., 2019) and a greater chance of burnout (Cook & Zill, 2021). Thus, workers with T1D may be a population that would benefit from greater health promotion efforts.

## Methods

### Study Overview

We analyzed a subset of participants (n = 92) that reported working full-time or part-time, from an EMA study investigating the between and within-person relationships between function, emotion, and blood glucose in adults with type 1 diabetes (T1D) (Pyatak et al., 2021). An in depth description of the study procedures is available in the corresponding protocol paper (Pyatak et al., 2021). Participants were recruited from three clinical sites through provider referrals, mail, email, and flyers. Inclusion criteria included being 18 years of age or older, and having a T1D diagnosis for more than a year. Participants also completed a baseline survey battery, 14 days of EMA data collection during which they wore a continuous glucose monitor (CGM), and a follow-up survey battery. Contents of the baseline and follow-up survey batteries included questionnaires assessing diabetes management, emotional well-being, functioning, basic demographics, and clinical information (e.g., method of insulin delivery). The majority of participants completed study procedures remotely, including training in the study protocol via videoconferencing. This training was completed in a single session with a trained research coordinator, and included practice in using the study smartphone, explanation of EMA items that were unclear, and instruction on how to wear the provided CGM. All the information provided by the research coordinators were also available in guidebooks that were sent to participants, in printed and/or PDF form, prior to the training call. The measures utilized in the present analysis were administered during the EMA portion of the study, in which up to 6 momentary surveys per day were administered at 3-hour intervals on study-provided smartphones. The data collection procedures were approved by the University of Southern California’s Institutional Review Board. Subjects provided informed consent to participate in the study electronically through an e-consent framework.

### Measures

#### Activity

Momentary activities were assessed with a single multiple-choice item shown in Table 1. The question and response options were presented simultaneously to participants on their phone screens at the beginning of every EMA survey. The list of activities was based on a taxonomy of activities created by occupational therapists based on their practice framework (American Occupational Therapy Association, 2020). Activity examples shown in Table 1 were presented in the guidebook used as training material for each participant; they were not shown in the EMA surveys to minimize on-screen text. Sleeping/napping was presented as one item in EMA surveys, but in analyses we anticipated that sleeping and napping may have different relationships with other variables. Whenever sleeping/napping was reported on the first survey of the day, which was typically close to time of waking, we assumed that the participant had just engaged in sleeping. Reporting sleeping/napping in EMA surveys during other parts of the day were assumed to be indicative of napping.

**Table 1:** Ambulatory assessment measures

Construct	Item(s)	Response Option(s)
**ACTIVITY**		
Activity type	What were you doing right before starting this survey? (Participants choose the one option that best applies.)	● Work/school activities (e.g. paid labor, volunteer work, and studying) ● Traveling (e.g. driving, riding in a car, walking) ● Relaxing/chilling (e.g. passive leisure like watching Netflix, listening to music) ● Sleeping/napping (separated in analyses) ● Socializing (e.g. talking with friends/family) ● Caring for myself (e.g. eating, dressing, bathing, toileting, personal grooming) ● Caring for others (e.g. caring for your children and pets, if you’re caring for others as part of work this counts as “work”) ● Doing housework/errands (IADLs) (e.g. paying bills, washing dishes and clothes, exercising for health) ● Fun/play/leisure activities (e.g. active leisure like exercising for fun, video games, reading for fun) ● Other (If chosen, please specify) NOTE: Activity examples were not in the actual item, but were explained during training and listed in a manual provided to participants
**HEALTH RELATED QUALITY OF LIFE RELEVANT MEASURES**		
**Mental Health**		
Stress	How stressed are you right now?	0 (Not at all stressed) to 100 (Extremely stressed)
Diabetes Stress	How stressed do you feel about your diabetes or diabetes management right now?	0 (Not at all stressed) to 100 (Extremely stressed)
Positive affect	Average of mood ratings for “happy”, “content”, “enthusiastic”, “excited”	For each mood, 0 (not at all) to 100 (extremely)
Negative affect	Average of mood ratings for “tense”, “upset”, “sad”, “disappointed”	For each mood, 0 (not at all) to 100 (extremely)
**Other Health Related Quality of Life Relevant Measures**		
Blood glucose	Measures passively with a continuous glucose monitor	N/A
Fatigue	At this moment, how tired do you feel?	0 (Not at all) to 100 (Extremely)
Activity Satisfaction	How satisfied are you with the way you did this activity?	0 (Not satisfied at all) to 100 (Extremely satisfied)
Activity Importance	How important is this activity to you?	0 (Not important at all) to 100 (Extremely important)

IADLs = instrumental activities of daily living

#### Other Ambulatory Assessment Measures

The other ambulatory assessment measures listed in Table 1 were used as HRQOL relevant metrics. EMA items were grouped under the broad categories of “mental health” and “other HRQOL relevant measures”. Mental health was a dimension of the SF-36 (Simon et al., 1998). For the “other HRQOL relevant measures”, blood glucose (BG) fits under the “biological function” section of Ferrans and colleagues’ HRQOL model (Ferrans et al., 2005). Fatigue fits under “symptoms”, and activity satisfaction under “functional status” in the same model. Activity importance may not be explicitly represented in the SF-36 or the Ferrans’ HRQOL model, but it was included as a HRQOL relevant measure because of prior research suggesting an association between meaningful activity engagement and QOL (Eakman et al., 2010; Goldberg et al., 2002).

All EMA items were asked after the initial activity question, and were derived from validated global measures and/or used successfully in previous EMA studies. Items addressing positive and negative affect were taken from the “Stress and Working Memory” (SAWM) study (Scott et al., 2018). The item for fatigue was used in a prior EMA study (Broderick et al., 2009), as were the questions on stress (Dunton et al., 2018) and diabetes distress (Merwin et al., 2015). Questions about activity satisfaction and importance were based on items in the Canadian Occupational Performance Measure (Law et al., 1990). All items had response options in a slider scale format with possible responses ranging from 0 (left anchor) to 100 (right anchor), a response format often used in EMA research (Buysse et al., 2007; Kikuchi et al., 2015; Schwartz et al., 2016).

BG was assessed with the Abbott FreeStyle LibrePro Flash Glucose Monitoring System. The device recorded interstitial glucose values every 15 min, and an algorithm was applied to these measures to estimate BG. To use the updated Libre2 algorithm, the continuous glucose monitors were sent to the Abbott Diabetes Scientific Research Group, who applied the algorithm and sent back data files with the BG estimates. Momentary values of BG between 70 and 180 mg/dL are considered in range, below 70 low, and above 180 high (Beck et al., 2019). Each EMA prompt was accompanied by a BG value recorded within 15 min prior to the prompt. In analyses, BG values were treated as continuous, and the cutoffs for low and high BG ranges specified above were used to aid with interpretation of results.

### Statistical Analyses

Multilevel structural equation modeling (MSEM) was used to examine the within- and between-person associations between activity engagement and HRQOL relevant variables, using the software M*plus* (Muthén & Muthén, 1998) via the R package MplusAutomation (Hallquist & Wiley, 2018). Two-level MSEM was used with EMA measurement occasions nested in individuals, and Bayesian parameter estimation. In separate models, each HRQOL variable was regressed on dummy coded variables indicating the type of activity reported on an EMA survey at level one (within people). At level two (between people), the latent average of a HRQOL variable was regressed on the latent average of each of the activity type variables. Following best practices for centering categorical predictors, the binary activity engagement dummy variables were person-mean centered at the within level, their latent averages were estimated at the between level, and fixed rather than random slopes were specified (Yaremych et al., 2021). Specifying random slopes, one for each regression of a HRQOL variable on an activity, would have been ideal. However, given the number of activity types, this would have resulted in a complex model with many correlated (between-person) random effects that would only be feasible with a large sample size. Because of our relatively small sample size of n = 92, we opted to use fixed instead of random slopes.

HRQOL variables were adjusted by time of day and day of week at level one, and demographic variables (i.e. age, gender, ethnicity, and income) at level two. Time of day was coded as a categorical variable indicating if the survey was the first, last, or one of the middle surveys of the day. This approach was taken to account for individual differences in sleep/wake times, as well as to account for the possibility that relationships between time of day and the HRQOL metrics were not linear. Both time of day and day of week were dummy coded, and person-mean centered at the within level. Fixed (instead of random) slopes were used for regressions of HRQOL variables on time of day and day of week at level one, again because of sample size concerns.

Efforts were made to facilitate the interpretability of estimates from the MSEM models. Level one estimates from the MSEM models (with HRQOL relevant metrics regressed on activity) indicated the mean difference in a HRQOL measure, within a person, between when a particular activity was reported and when “relaxing/chilling” (reference group) was reported. “Relaxing/chilling” was chosen as the reference category because it was the most frequently indicated activity, and was also most similar to a baseline state, thereby making comparisons to other activities more intuitive. The level one unstandardized estimates were divided by the level one standard deviation of the HRQOL variable, following one form of multilevel standardization in M*plus* (Muthén & Asparouhov, 2010). Using this standardization, within-person estimates represent the size of differences from the reference group in standard deviations of the HRQOL variable, and their magnitude can be interpreted with Cohen’s *d* effect size guidelines where differences of 0.2 indicate small, 0.5 moderate, and 0.8 large effects, respectively (Cohen, 2013). Unstandardized BG estimates had meaningful interpretations, so these were reported in addition to their standardized estimates.

When an outcome is regressed on a categorical variable in a multilevel model, the between-person estimate of a particular level of a categorical variable can be interpreted as the mean difference in the outcome variable, when comparing an individual who engaged in the reference activity all of the time (i.e., people who only reported “relaxing/chilling”) to an individual who engaged in the focal category all of the time (e.g. people who only reported “caring for others”) (Yaremych et al., 2021). This interpretation may not be intuitive because it is unlikely for an individual to only engage in a single activity during the entire study period. To improve interpretability, we followed the recommendation by Yaremych et al. (2021) and divided the between-person regression coefficients by 10 (Yaremych et al., 2021). Such a procedure allows interpretation in terms of a linear increase, and captures the mean difference between the categorical variable level of interest and the reference group, if the individual engaged in the activity 10% of the time (e.g. a person reported “caring for others” 10% of the time). If activity engagement was instead 20% of the time, the mean difference would double, triple for 30% of the time, etc.

Analogous to the standardization for level one regression coefficients, level two coefficients were also divided by the level two standard deviation of the HRQOL variable. After these transformations, between-person coefficients represented the mean difference (in standard deviations) in the HRQOL variable, when comparing a person that only reported “relaxing/chilling” to another person who reported engaging in the specific activity of interest (e.g. “caring for others) 10% of the time.

For activities with between-person aspects that were strongly associated with HRQOL relevant measures, we further examined how the average frequencies of engagement in those activities were related to relevant participant characteristics. This was done using two-level models where the activity of interest was specified as a categorical dependent variable, and the random intercept (Level 2) of the activity variable was regressed on one participant characteristic at a time with a logit linkage. Regression estimates from these models represented how participant characteristics predicted the log odds that the activity of interest was reported, across all measurement events for a person. For ease of interpretability, the log odds estimates were exponentiated so that they could be represented as odds ratios, for which 95% confidence intervals that do not contain 1 indicate statistical significance. For continuous predictors, exponentiated log odds estimates indicated the factor by which the probability of the outcome changed for every one unit change in the predictor.

Instead of adjusting for the multiple statistical tests with statistical corrections of the alpha level, we reported 95% confidence intervals for the parameters from all statistical tests. This approach has been argued to reduce the chance of type II error, by avoiding the often overly conservative statistical corrections of alpha (Rothman, 1990). To reduce the chance of type I error, readers will need to informally account for multiple comparisons when interpreting results in part by looking at the confidence intervals.

### Statistical Power

Monte Carlo simulations in Mplus (Muthén & Muthén, 1998) were used to examine the magnitude of effects detectable with our sample size. With n = 92, approximately 70 momentary observations per person collected over the two weeks, and for an activity reported 15% of the time, there was 80% power to detect a standardized mean difference (Cohen’s d) of 0.097 in the outcome variable when comparing an activity and the reference group. On the between-person level, assuming an intraclass correlation (ICC) of 0.5 for an outcome variable, a sample of n = 92 has 80% power to detect a moderate correlation (r = 0.29) between the proportion of time an activity was reported and between-person variation of an outcome.

## Results

Analyses were conducted using data from a total of 92 participants that identified as workers; Table 2 shows the demographic characteristics of our sample. Participants were not required to indicate their occupations, but examples of jobs reported included cashier, lawyer, housekeeper, teacher, security guard, and physician. The mean of the individual-specific average work hours reported (i.e., average of the average for each cluster), on workdays, was 4.82 h (SD = 2.23). Across all participants, four or more surveys were completed on 82% of days. The median EMA completion percentage was 91%.

**Table 2:** Demographics

Characteristic	n	Mean (SD) or Percentage (%)
Age (years)	92	39.8 (14.4)
Gender		
Male	43	47%
Female	49	53%
Ethnicity		
White	31	34%
Latino	32	35%
African American	17	18%
Multi-ethnic	4	4%
Asian	3	3%
Other	2	2%
Not reported	3	3%
Preferred Language		
English	82	89%
Spanish	10	11%
Employment status		
Full-time	69	75%
Part-time	23	25%
Education		
High school grad or less	17	18%
Some college	27	29%
Bachelor’s degree	30	33%
Graduate degree	17	18%
Not provided	1	1%
Annual household income		
<$25,000	9	10%
$25,000-$49,999	26	28%
$50,000-$74,999	9	10%
≥$75,000	29	32%
Not provided	19	21%

### Within-Person Relationships Between Activity Engagement and HRQOL

Table 3 shows the standardized within-person estimates from MSEM, which indicate the average within-person difference for each activity from reference group “relaxing/chilling”. Their magnitudes can be evaluated based on Cohen’s *d* guidelines of 0.2 for small, 0.5 for medium, and 0.8 for large effects. The three activities associated with the greatest amount of stress were “caring for others,” d = 0.49, CI = [0.23, 0.64], “work/school activities,” d = 0.40, CI = [0.32, 0.48], and “traveling,” d = 0.34, CI = [0.19, 0.45]. These activities also had the highest mean negative affect ratings within a person. “Napping” was associated with the lowest level of diabetes distress, d = -0.20, CI = [-0.34, -0.09], while sleeping was associated with the lowest level of blood glucose, d = -0.27, CI = [-0.43, -0.10]. Within a person, activities associated with the highest level of positive affect were “socializing,” d = 0.36, CI = [0.24, 0.52], and “fun/play/leisure,” d = 0.35, CI = [0.22, 0.47], while the lowest average positive affect levels were for “caring for others,” d = -0.22, CI = [-0.41, -0.08], “sleeping,” d = -0.25, CI = [-0.37, -0.08], and “napping,” d = -0.37, CI = [-0.51, -0.25]. Fatigue was on average higher after having just reported “napping,” d = 0.23, CI = [0.11, 0.34], and “sleeping,” d = 0.25, CI = [0.10, 0.41]. Participants were typically least satisfied with their performance of “napping,” d = -0.45, CI = [-0.62, -0.33], and “sleeping,” d = -0.41, CI = [-0.55, -0.27]. The greatest momentary ratings of activity importance were given for “sleeping,” d = 0.82, CI = [0.67, 0.95], and “caring for others,” d = 0.68, CI = [0.52, 0.86]. Lowest activity importance ratings were for “fun/play/leisure,” d = -0.27, CI = [-0.43, -0.17], and “relaxing/chilling” (reference group).

**Table 3:** Within-person (level one) standardized (on the dependent variable) regression coefficients and corresponding 95% confidence intervals. Estimates were from multilevel models with activity type as the predictors and various quality of life relevant measures as the dependent variable at levels one and two. The standardized estimates represent the average within-person difference from reference group “relaxing/chilling”, as measured by standard deviations of the HRQOL relevant variable. Estimates can be interpreted according to Cohen’s *d* effect size guidelines (0.2 small, 0.5 moderate, and 0.8 large)

	N obs	Stress	Diabetes distress	Positive affect	Negative affect	Fatigue	Blood glucose	Blood glucose (mg/dL)	Activity satis.	Activity import.
Relaxing/ chilling	1251	Reference group						103.83		
Caring for myself	587	0.13 (0.01, 0.21)*	0.06 (-0.05, 0.14)	-0.04 (-0.17, 0.06)	0.1 (-0.02, 0.19)	-0.12 (-0.23, -0.03)*	-0.13 (-0.25, -0.01)*	96.11	0.04 (-0.06, 0.13)	0.42 (0.29, 0.5)*
Caring for others	119	0.49 (0.23, 0.64)*	-0.08 (-0.28, 0.13)	-0.22 (-0.41, -0.08)*	0.47 (0.21, 0.62)*	0.09 (-0.1, 0.28)	0.05 (-0.2, 0.25)	106.49	0.07 (-0.15, 0.23)	0.68 (0.52, 0.86)*
Housework/ errands	604	0.19 (0.09, 0.27)*	-0.05 (-0.15, 0.05)	-0.02 (-0.13, 0.09)	0.1 (0, 0.19)*	-0.22 (-0.32, -0.12)*	-0.02 (-0.13, 0.09)	102.9	-0.02 (-0.11, 0.08)	0.04 (-0.07, 0.15)
Fun/play/ leisure	351	-0.2 (-0.31, -0.09)*	-0.03 (-0.16, 0.1)	0.35 (0.22, 0.47)*	-0.18 (-0.29, -0.06)*	-0.17 (-0.28, -0.08)*	-0.1 (-0.24, 0.05)	99.19	0.02 (-0.09, 0.15)	-0.27 (-0.43, -0.17)*
Napping	339	-0.02 (-0.14, 0.09)	-0.2 (-0.34, -0.09)*	-0.37 (-0.51, -0.25)*	-0.07 (-0.2, 0.04)	0.23 (0.11, 0.34)*	-0.02 (-0.15, 0.14)	104.45	-0.45 (-0.62, -0.33)*	0.18 (0.04, 0.31)*
Sleeping	473	-0.01 (-0.17, 0.15)	-0.06 (-0.21, 0.07)	-0.25 (-0.37, -0.08)*	0.04 (-0.12, 0.21)	0.25 (0.1, 0.41)*	-0.27 (-0.43, -0.1)*	86.7	-0.41 (-0.55, -0.27)*	0.82 (0.67, 0.95)*
Socializing	231	-0.05 (-0.16, 0.13)	-0.02 (-0.17, 0.1)	0.36 (0.24, 0.52)*	0.09 (-0.03, 0.26)	-0.39 (-0.56, -0.27)*	0.09 (-0.05, 0.24)	111.75	0.08 (-0.07, 0.18)	0.14 (-0.01, 0.29)
Traveling	340	0.34 (0.19, 0.45)*	0.06 (-0.06, 0.19)	0.07 (-0.06, 0.22)	0.24 (0.09, 0.35)*	-0.01 (-0.12, 0.12)	0.05 (-0.1, 0.2)	109.6	0.01 (-0.15, 0.14)	0.02 (-0.11, 0.15)
Work/ school activities	1893	0.4 (0.32, 0.48)*	0.02 (-0.06, 0.09)	-0.13 (-0.22, -0.05)*	0.22 (0.14, 0.3)*	-0.11 (-0.19, -0.03)*	-0.06 (-0.15, 0.03)	100.25	-0.18 (-0.26, -0.09)*	0.28 (0.19, 0.36)*

*Confidence interval does not contain 0, indicating statistical significance

import.=importance; satis.=satisfaction

### Between-Person Relationships Between Activity Engagement and HRQOL

Table 4 shows the standardized between-person regression coefficients from MSEM. They indicate the difference in average levels of HRQOL metrics, in standard deviations of the HRQOL metric, when comparing a person that reported the activity indicated in the rows 10% of the time, to another hypothetical person that only reported engaging in “relaxing/chilling” on all EMAs over the study period. For each 10% more time that a person reported “caring for others”, their average level of stress was 0.99 SDs higher, CI = [0.27, 1.57], diabetes stress was 1.03 SDs higher, CI = [0.44, 1.77], and negative affect was 0.85 SDs greater, CI = [0.06, 1.45]. For each 10% more time that a person indicated caregiving, average blood glucose was on average 0.72 SDs higher, CI = [0.04, 1.36], or 46.19 mg/dL greater. Those that indicated “napping” 10% more of the time had blood glucose that was on average 0.54 SDs higher, CI = [0.21, 0.86], or 34.52 mg/dL higher. Participants that reported more “napping” also on average experienced greater stress (ß =0.49, CI = [0.13, 0.84]), diabetes stress (ß =0.68, CI = [0.36, 1.02]), negative affect (ß =0.53, CI = [0.20, 0.92]), and lower activity satisfaction (ß =-0.41, CI = [-0.75, -0.07]). Finally, for each 10% more time that a person reported “sleeping”, their average level of fatigue was 0.65 SDs higher, CI = [0.10, 1.21], and average activity satisfaction was 0.63 SDs lower, CI = [-1.31, -0.02].

We further examined whether the frequencies of napping and caregiving were associated with participant characteristics. Gender (OR = 0.85, CI= [0.21, 3.50]), number of years with T1D (OR = 0.97, CI= [0.90, 1.05]), age (OR = 0.97, CI= [0.92, 1.03]), number of self-reported diagnoses (OR = 1.35, CI= [0.79, 2.30]), and average blood glucose in mg/dL (OR = 1.00, CI= [0.99, 1.02]) were not significantly associated with napping frequency. A greater percentage of time in an optimal blood glucose range (70 to 180 mg/dL) was associated with decreased napping frequency (OR = 0.96, CI= [0.92, 0.99]). Put another way, for every 1% increase in time spent with optimal blood glucose, napping frequency changed by a factor of 0.96. In terms of caregiving, males engaged in caregiving less frequently (OR = 0.14, CI= [0.03, 0.72]) compared to females.

**Table 4:** Between-person (level two) regression coefficients (and corresponding 95% confidence intervals) from mixed models with activity type as the predictors and various quality of life relevant measures as the dependent variable at levels one and two. The estimates represent the difference in average levels of HRQOL metrics, in standard deviations of the HRQOL metric, when comparing a person that reported the activity indicated in the rows 10% of the time, to another hypothetical person that only reported relaxing/chilling. If activity engagement was instead 20% of the time, the difference would double, triple for 30% of the time, etc.

	N obs	Stress	Diabetes distress	Positive affect	Negative affect	Fatigue	Blood glucose	Blood glucose (mg/dL)	Activity satis.	Activity import.
Relaxing/ chilling	1251	Reference group								
Caring for myself	587	0.02 (-0.26, 0.36)	0.12 (-0.22, 0.4)	0.24 (-0.1, 0.55)	0.08 (-0.17, 0.45)	0 (-0.4, 0.32)	-0.08 (-0.38, 0.24)	-4.93	0.06 (-0.29, 0.35)	0.19 (-0.28, 0.47)
Caring for others	119	0.99 (0.27, 1.57)*	1.03 (0.44, 1.77)*	0.05 (-0.84, 0.68)	0.85 (0.06, 1.45)*	0.29 (-0.28, 0.89)	0.72 (0.04, 1.36)*	46.19	-0.64 (-1.46, 0.08)	0.07 (-0.87, 0.72)
Housework/ errands	604	0.01 (-0.35, 0.33)	-0.16 (-0.55, 0.28)	-0.3 (-0.77, 0.18)	-0.03 (-0.4, 0.3)	0.2 (-0.23, 0.64)	-0.13 (-0.54, 0.27)	-8.16	-0.07 (-0.42, 0.43)	-0.02 (-0.52, 0.53)
Fun/play/ leisure	351	-0.12 (-0.38, 0.13)	-0.1 (-0.34, 0.13)	0.1 (-0.15, 0.39)	-0.08 (-0.32, 0.22)	0.07 (-0.19, 0.33)	-0.03 (-0.27, 0.18)	-1.93	-0.02 (-0.31, 0.21)	-0.12 (-0.41, 0.18)
Napping	339	0.49 (0.13, 0.84)*	0.68 (0.36, 1.02)*	0.12 (-0.16, 0.55)	0.53 (0.2, 0.92)*	0.3 (-0.03, 0.63)	0.54 (0.21, 0.86)*	34.52	-0.41 (-0.75, -0.07)*	-0.11 (-0.37, 0.25)
Sleeping	473	0.31 (-0.26, 0.84)	0.3 (-0.26, 0.83)	0.13 (-0.42, 0.55)	0.23 (-0.32, 0.85)	0.65 (0.1, 1.21)*	0.3 (-0.35, 0.83)	19.27	-0.63 (-1.31, -0.02)*	-0.14 (-0.64, 0.47)
Socializing	231	0.14 (-0.4, 0.66)	0.16 (-0.33, 0.59)	-0.02 (-0.6, 0.48)	-0.07 (-0.56, 0.53)	0.32 (-0.2, 0.76)	0.1 (-0.28, 0.55)	6.64	-0.07 (-0.64, 0.42)	0.12 (-0.38, 0.67)
Traveling	340	0.16 (-0.21, 0.46)	0.23 (-0.09, 0.51)	-0.05 (-0.41, 0.38)	0.07 (-0.36, 0.36)	0.02 (-0.28, 0.32)	0.08 (-0.24, 0.36)	4.98	-0.09 (-0.4, 0.22)	-0.09 (-0.49, 0.2)
Work/ school activities	1893	0.17 (-0.07, 0.35)	0.09 (-0.13, 0.31)	0.15 (-0.12, 0.46)	0.13 (-0.07, 0.33)	0.12 (-0.08, 0.32)	0.21 (-0.02, 0.4)	13.58	-0.06 (-0.32, 0.27)	0.04 (-0.31, 0.36)

*Confidence interval does not contain 0, indicating statistical significance

import.=importance; satis.=satisfaction

## Discussion

MSEM was used to examine the within- and between-person associations between activity engagement and HRQOL relevant measures in adults with T1D, to provide a quantitative representation of the lived experience of T1D. A multitude of significant relationships were uncovered, but below we focus on interpreting those with greatest potential for informing health promotion efforts. We also emphasize associations that differed across within and between-person levels.

“Caring for others” was associated with lower HRQOL at both the within and between-person levels, suggesting that workers with T1D who are also caregivers may especially require health promotion attention. At the momentary level, “caring for others” was linked with increased stress, negative affect, and lower positive affect. At the between-person level, individuals who, on average, engaged more frequently in “caring for others” had higher stress, diabetes distress, negative affect, and mean BG. Notably, a person that reported “caring for others” 20% of the time would have an average BG of approximately 103.83(intercept) + 46.19(caring for others unstandardized estimate)*2 = 196.21 mg/dL, which is in the high BG range (Beck et al., 2019). Although power at the between-person level was limited due to the relatively small number of reports of “caring for others”, a relationship with BG was still evident. Decreased QOL associated with “caring for others” may originate from needing to cope with the three major demand sources of work, diabetes self-management (Fisher et al., 2015), and caregiving (Dich et al., 2015). It is also noteworthy that “caring for others” was associated with one of the highest levels of momentary ratings of activity importance. Thus, the momentary decrements in some HRQOL metrics (e.g. emotional health) associated with “caring for others” may to some extent be counterbalanced by a high level of meaning and importance ascribed to the task. Engagement in meaningful activities has been associated with greater perceived QOL (Goldberg et al., 2002). Finally, women were more likely than men to report engaging in “caring for others”, consistent with prior literature (Revenson et al., 2016). Women may therefore be more likely than men to experience decreased HRQOL from caregiving activities.

Results suggested that excessive “napping” (i.e. reporting napping 10% or more of the time during a person’s waking hours) may be a sign that a working individual with T1D may benefit from health promotion efforts. At the momentary level, there was little evidence that napping was detrimental to HRQOL. For example, reports of napping were associated with higher fatigue, but also relatively low stress and diabetes stress compared to other activities. Its association with greater fatigue and lower positive affect at the within-person level may have been influenced by the EMA sampling strategy used. In reporting napping, there is a chance that people were woken up in the middle of napping by a loud phone notification to complete the EMA survey. Between people, individuals that engaged in napping more frequently experienced higher average levels of stress, diabetes stress, negative affect, and BG, and lower average activity satisfaction. A person that reported “napping” 25% of the time would have an average BG of approximately 103.83(intercept) + 34.52(napping unstandardized estimate)*2.5 = 190.13 mg/dL, which is in the high BG range (Beck et al., 2019). This finding is consistent with prior research suggesting that excessive daytime napping may be a sign of underlying health risk (Leng et al., 2014; Xiao et al., 2017), and one study that found an association between higher napping frequency and lower glycemic control (Bawadi et al., 2021). In follow up analyses with other health risk metrics, we found that greater napping frequency was not significantly associated with the number of reported diagnoses or average BG, but was associated with decreased time in optimal BG range. Thus, the underlying health risk metric of time in optimal BG range may have particular relevance to napping frequency. In terms of other participant characteristics, no significant associations were found between napping frequency and gender or age, in contrast to prior literature (Inazumi et al., 2020).

Within individuals, “sleeping” was associated with lower levels of satisfaction relative to other activities, but also had the highest rating of importance, suggesting that it may also be worth addressing in health promotion for workers with T1D. One reason why sleeping may have been associated with lower activity satisfaction ratings is because all adults in our sample had type 1 diabetes, a population that often experiences poorer sleep as compared to people without the condition (Farabi, 2016). Even among workers without diabetes, though, occasional difficulties with sleep are common (Knudsen et al., 2007). The high importance ratings may suggest that most individuals in our sample understood the importance of sleep but still often had difficulty sleeping, or that the experience of poor sleep increased the perception of its importance. Between people, individuals that engaged in sleeping more frequently typically experienced more fatigue and lower average activity satisfaction. Perhaps people with sleeping difficulties spend more time attempting to sleep, but still feel fatigued, leading to lower activity satisfaction overall.

Finally, work/school had a small to moderate association with stress at the within-person level, and no associations with HRQOL variables at the between-person level. One reason for the lack of association with HRQOL metrics may be that the mean of the individual-specific average work hours reported (i.e., average of the average for each cluster), on workdays, was 4.82 h (SD = 2.23). Prior literature has found that working more than ten hours daily was associated with lower mental and physical health (Bannai & Tamakoshi, 2014; Wong et al., 2019).

### Health Promotion Implications

Collectively, study results provide a quantitative representation of the lived experience of T1D that could inform the clinical reasoning of practitioners of the Lifestyle Redesign® (LR) approach. For instance, average BG was found to be associated with frequencies of both napping and caregiving. With this in mind, for clients frequently engaged in napping or caregiving, LR practitioners may consider evaluating whether and how these activities relate to their diabetes management. If a client struggles to manage his/her diabetes in part because of responsibilities from caregiving, then part of the intervention may entail brainstorming strategies to carry out both duties so that they conflict less with one another. As another example, at the momentary level, “relaxing/chilling” and “fun/play/leisure” were typically associated with the lowest activity importance ratings. If a client is not engaging in optimal diabetes management because he/she is burned out from the collective load from responsibilities such as health management, part of an intervention may entail reinforcing the importance of relaxation/leisure, and/or brainstorming forms of relaxation/leisure aligned with the client’s values.

### Limitations

Limitations regarding statistical power should be noted. Power to detect associations between the various activity types and HRQOL relevant metrics varied as a function of the number of times activities were reported. For activity types less frequently reported (such as “caring for others” and “socializing” in this sample), a larger number of individuals may need to be sampled to capture small yet potentially meaningful associations. Furthermore, our sample of n = 92 and approximately 70 observations per person was sufficiently powered to detect small associations at the within-person level, but only moderately strong associations at the between-person level. Thus, small but possibly meaningful between-person associations between activity engagement frequency and HRQOL variables may have remained undetected. Finally, with only 92 individuals, specifying random slopes between each activity and a HRQOL variable at level one was infeasible. Our approach of using only random intercepts assumed that all individuals had the same associations between activity engagement and HRQOL. Future studies with larger sample sizes are needed to examine if our results replicate when using more complex models with random slopes, which allow for different associations between activity and HRQOL for each individual.

Additional evidence may be needed to demonstrate that our results apply to a general population of adults. Data collection was completed during various stages of the COVID-19 pandemic, during which participants were likely faced with several changes including social distancing requirements. This context may have affected the patterns and nature of activity engagement reported, and their associations with HRQOL variables. For instance, “socializing” may have entailed videoconferencing rather than in-person meetings, which may have affected its relationship with HRQOL.

Another possible limitation is the potential lack of precision of the EMA activity item. While we were able to attain meaningful results with the broad categories used, more specific activity choices (e.g. types of relaxing/chilling like watching television or meditating, or separate category for different kinds of exercise) may have helped increase the specificity of activities in each category. In future studies, one potentially useful approach is asking participants about activities in a two-step process. A first step could identify the general category that best fits the task just engaged in; after that, branching logic could be used to ask the participant to choose from a more specific list of activities belonging to the general category chosen, allowing for greater precision while minimizing participant burden.

Finally, we could not infer directionality of the relationships between activity engagement and HRQOL measures. For instance, we could not conclude whether greater frequency of napping contributed to suboptimal BG levels, or if suboptimal BG levels contributed to a greater frequency of napping. Data on activity type and HRQOL metrics were analyzed contemporaneously. While it would have been possible to examine lagged effects between activities and HRQOL at the momentary level, there were at least three hours between surveys, and we expected associations among activity engagement and HRQOL to occur much faster than that. Thus, the analysis of lagged effects would not have been consistent with our conceptual model.

## Conclusions

Analyzing the between- and within-person relationships between activity engagement and HRQOL may provide insights potentially useful for health promotion efforts, as was demonstrated here in a population of workers with T1D. Results of this study suggested that workers with T1D that also engage in caregiving duties frequently may be at greater risk for suboptimal BG levels, and hence may require greater attention in health promotion efforts. Napping appeared to only be problematic when engaged in excessively (e.g., reporting participating in it 25% of the time or more during waking hours), as it also coincided with suboptimal BG. However, we could not speak to whether excessive napping was a precedent or antecedent to a greater amount of time on average with suboptimal BG levels. Sleep was associated with low satisfaction ratings, and may be an important activity to target in health promotion efforts for workers with T1D. Examining the between and within-person relationships between activity engagement and HRQOL may also be fruitful for other populations with chronic conditions.

## Electronic Supplementary Material

Below is the link to the electronic supplementary material.

Supplementary Material 1

## Data Availability

The data that support the findings of this study are available from the corresponding author, R.H., upon reasonable request.

## Code Availability

The software code that supports the findings of this study is available from the corresponding author, R.H., upon reasonable request.
