# Supplementary material for: Across and Within- Individual Associations Between Everyday Activities and Quality of Life Relevant Measures, in Workers with Type 1 Diabetes
Source: Appl Res Qual Life. 2023 Apr 20:1–23. Online ahead of print. doi: 10.1007/s11482-023-10171-2 (PMC10116107; doi:10.1007/s11482-023-10171-2)
Supplement: Supplementary file 1 — Supplementary Material 1 [file 11482_2023_10171_MOESM1_ESM.docx]

Responses to Reviewers

We thank the reviewer for the thoughtful feedback, which was helpful for strengthening the manuscript. Our responses to the comments are below.

INTRODUCTION
*       P. 2: In the first paragraph, I suggest providing a more comprehensive definition of HRQOL in the 2nd sentence. From the 2nd sentence, it is unclear how HRQOL differs from perceived health.

The definition was updated to the below. This definition still has a large degree of overlap with perceived health, but it was most consistent with how HRQOL was assessed in this paper.

“Various definitions and models of HRQOL exist (Bakas et al., 2012), but here we use its conceptualization as “how well a person functions in their life and his or her perceived wellbeing in physical, mental, and social domains of health” (Hays & Reeve, 2008, p.195).”

*       P. 2: If the SF-36 is an abbreviation for something, please provide the full name before the first use of the abbreviation.

The full name is now provided (Short Form Health Survey).

*       Before the "present study" section, the authors should provide more justification for this study of adults with T1D specifically. From the current writing, it is not completely clear how this within- and between-person analysis could offer new insight into the lived experience of adults with T1D. It is also not clear how such an analysis could or should inform health promotion efforts. Please clarify how and why this study is important.

The text below was added before the “present study” section.

“Adults with type 1 diabetes (T1D) represent one population that may benefit from investigations of the between- and within-person and relationships between activity engagement and HRQOL. T1D is characterized by destruction of beta cells in the pancreas, leading to absolute insulin deficiency (Kerner & Brückel, 2014). Individuals with T1D take on several tasks to manage their condition including checking blood glucose, engaging in regular physical activity, planning timing and content of meals, and regular dosing of insulin (J. Beck et al., 2017). Both health promotion efforts for T1D, and investigations of the lived experience of T1D, often focus on these specific self-management behaviors (Hansen et al., 2018; Hood et al., 2010; King et al., 2017; Quirk et al., 2014). However, from a Lifestyle Redesign® (LR) perspective (Pyatak et al., 2022), all daily activities can be impacted by T1D, and in turn affect overall health and well-being.

Examination of the between- and within-person relationships across a wide spectrum of activity engagements and HRQOL in adults with T1D may be particularly relevant for LR, an intervention framework that “promotes awareness of the relationship between everyday activities and health and guides people in the process of orchestrating occupations, habits, and routines to enhance health and well-being (Pyatak et al., 2022).” Interventions based on LR have been shown to improve the health and quality of life of various populations including adults with diabetes, chronic pain, and multiple sclerosis (Pyatak et al., 2022). Knowledge of the relationships between daily activity engagements and HRQOL could be particularly beneficial for this intervention framework, given its focus on the orchestration of daily activities for optimal well-being.”

*       Later in the paper (top of P. 8), the authors describe aspects of Ferrans and colleague's model of HRQOL. To an unfamiliar reader, this is unclear. I suggest expanding more on this theoretical model of HRQOL in the introduction.

The below text was added to the first paragraph of the introduction:

“According to Ferrans and colleagues’ HRQOL model, a multitude of factors can contribute to HRQOL (e.g., functional status, symptoms, and biological functioning), all of which are impacted by both characteristics of individuals and the environment (Ferrans et al., 2005).”

METHODS
*       P. 5: please indicate what CGM stands for before the first use of the abbreviation.

Thank you for catching this. We now indicate that CGM stands for continuous glucose monitor before its first use.

*       P. 5: The authors mentioned that participants completed a survey battery at baseline and follow-up, but detail is provided only about the surveys that were administered with EMA. Please provide more detail about the baseline and follow-up survey items; this can be brief if not included in the present analyses.

The sentence below was added to describe the baseline and follow up surveys, in the “study overview” section.

“Contents of the baseline and follow-up survey batteries included questionnaires assessing diabetes management, emotional well-being, functioning, basic demographics, and clinical information (e.g., method of insulin delivery).”

*       P. 5: More detail about study procedures should be added. It is mentioned that training was involved. Was this a single training session, or multiple? Later it is mentioned that a guidebook was provided; please provide more information about this. Were there other aspects of the study procedure that are not mentioned in the current writing?

We added additional information about the study procedure (shown below) under “study overview”.

“The majority of participants completed study procedures remotely, including training in the study protocol via videoconferencing. This training was completed in a single session with a trained research coordinator, and included practice in using the study smartphone, explanation of EMA items that were unclear, and instruction on how to wear the provided CGM. All the information provided by the research coordinators were also available in guidebooks that were sent to participants, in printed and/or PDF form, prior to the training call.”

For readers interested in additional details, we clarified that more comprehensive information was provided in a paper that provides an in-depth description of the study protocol.

“An in depth description of the study procedures is available in the corresponding protocol paper (Pyatak et al., 2021).”

*       Table 1: indicate what IADLs stands for.

We have clarified that IADLs stands for “instrumental activities of daily living” in the table notes.

*       Table 1 indicates that most self-report questions consisted of a 0-100 response set. Could the authors explain why this response set was chosen? I have rarely seen usages of this in other studies. For example, the positive/negative affect scale was said to follow the structure of the PANAS, but the PANAS was designed to have a 5-point Likert-type response set. Additionally, please note whether participants typed in a number or used a sliding scale.

0-100 response formats are commonly used in EMA studies, and we clarified this in text under “ambulatory assessment measures” (below). We also deleted the phrase stating that items followed the structure of the PANAS, as it is true that different response options were used. Finally, we clarified that participants used a slider scale (text below).

“All items had response options in a slider scale format with possible responses ranging from 0 (left anchor) to 100 (right anchor), a response format often used in EMA research (Buysse et al., 2007; Kikuchi et al., 2015; Schwartz et al., 2016).”

*       Was BG treated as a continuous or categorical (low/in range/high) variable? Later writing suggests it was treated as categorical, but this should be specified at the outset.

Thank you for pointing out the need for greater specificity here. In the last paragraph under “Other Ambulatory Assessment Measures” the below text was added.

“Each EMA prompt was accompanied by a BG value recorded within 15 minutes prior to the prompt. In analyses, BG values were treated as continuous, and the cutoffs for low and high BG ranges specified above were used to aid with interpretation of results.”

*       Statistical analysis:
o       In general, the authors do a good job of describing their analysis plan and the steps that were taken to ensure interpretability of parameter estimates. I commend the authors for their transformations enabling the coefficients to be interpreted in terms of effect size. I suggest adding these details:
o       Clarify why fixed rather than random slopes were specified.

The clarification below was added to the first paragraph under “statistical analyses”.

“Specifying random slopes, one for each regression of a HRQOL variable on an activity, would have been ideal. However, given the number of activity types, this would have resulted in a complex model with many correlated (between-person) random effects that would only be feasible with a large sample size. Because of our relatively small sample size of n=92, we opted to use fixed instead of random slopes.”

o       Were the level-1 categorical covariates (time of day and day of week) person-mean centered? If not, they should be. Person-mean centering some but not all level-1 predictors is likely to lead to biased estimates.

As recommended by the reviewer, we have adjusted the analyses and have person-mean centered level 1 categorical covariates. The text in the second paragraph under “statistical analyses” was updated accordingly (see below). Furthermore, Tables 3 and 4 were updated, and the description of those results was also revised.

“HRQOL variables were adjusted by time of day and day of week at level one, and demographic variables (i.e. age, gender, ethnicity, and income) at level two. Time of day was coded as a categorical variable indicating if the survey was the first, last, or one of the middle surveys of the day. This approach was taken to account for individual differences in sleep/wake times, as well as to account for the possibility that relationships between time of day and the HRQOL metrics were not linear. Both time of day and day of week were dummy coded, and person-mean centered at the within level. Fixed (instead of random) slopes were used for regressions of HRQOL variables on time of day and day of week at level one, again because of sample size concerns.”

*       Throughout the Methods and Results sections, the model described in the 3rd paragraph on P. 11 needs to be clarified. What is meant by a "multilevel dependent variable"? At one point the writing suggests that average frequency of engagement was used as the outcome variable, but in fact the outcome variable was binary. In multilevel logistic models the outcome may be binary, but the random intercept is continuous. So the effects of predictors on those random intercepts are linear slopes, not odds ratios.

Thank you for identifying the lack of clarity here. The text under “statistical analyses” was revised to the below.

“For activities with between-person aspects that were strongly associated with HRQOL relevant measures, we further examined how the average frequencies of engagement in those activities were related to relevant participant characteristics. This was done using two-level models where the activity of interest was specified as a categorical dependent variable, and the random intercept (Level 2) of the activity variable was regressed on one participant characteristic at a time with a logit linkage. Regression estimates from these models represented how participant characteristics predicted the log odds that the activity of interest was reported, across all measurement events for a person. For ease of interpretability, the log odds estimates were exponentiated so that they could be represented as odds ratios, for which 95% confidence intervals that do not contain 1 indicate statistical significance. For continuous predictors, exponentiated log odds estimates indicated the factor by which the probability of the outcome changed for every one unit change in the predictor.”

RESULTS
*       N = 92 seems like a very small sample size for MSEM. Was a power analysis conducted?

We added text describing results of statistical power analyses in the methods section, under “statistical power”.

“Monte Carlo simulations in Mplus (L. K. Muthén & Muthén, 1998) were used to examine the magnitude of effects detectable with our sample size. With n=92, approximately 70 momentary observations per person collected over the two weeks, and for an activity reported 15% of the time, there was 80% power to detect a standardized mean difference (Cohen’s d) of 0.097 in the outcome variable when comparing an activity and the reference group. On the between-person level, assuming an intraclass correlation (ICC) of 0.5 for an outcome variable, a sample of n=92 has 80% power to detect a moderate correlation (r=0.29) between the proportion of time an activity was reported and between-person variation of an outcome.”

*       When reporting confidence intervals, the following format is easier to follow as a reader, using the first result as an example: … "caring for others," d = 0.65, CI = [0.60, 0.71], …

The formatting of the confidence intervals was changed accordingly.

*       Table 2: I suggest reformatting so the confidence intervals are not broken up across lines. The authors could put the full confidence interval on a new line within each cell. (Same suggestion for Table 4.)

Tables 3 and 4 were reformatted accordingly.

*       P. 16: associations with participant characteristics were examined for napping and caregiving. There was also a significant result for work/school activities and blood glucose across persons; why wasn't this examined in terms of participant characteristics?
The significant association between work and BG across persons was smaller compared to the associations between BG and napping/caregiving. In any case, in the updated analyses, there was no longer a significant association between work/school activities and blood glucose at the between-person level.

DISCUSSION:
*       P. 21: the authors note that the minimal association between work/school and other outcomes may be due to the sample average of 6.77 work hours per day. However, the sample standard deviation was large enough that a significant proportion of the sample reported working 8 or more hours per day. This seems like a weak argument. I suggest framing this result differently.

Thank you for this comment. The sample average of 6.77 work hours per day was computed by pooling together all the workdays reported across participants, and finding the mean of the work hours reported. With this approach, individuals that worked more days during the study period were weighted more, as compared to individuals that worked less days. In discussions among the authors, we concluded that it would be more sensible to examine the individual- specific average work hours by instead computing average work hours reported on workdays for each person, and then averaging these across all people. Using this computation, we found that the average of mean work hours reported was 4.82 hours (SD=2.23). The final paragraph in the discussion section was revised to the below:

“Finally, work/school had a small to moderate association with stress at the within-person level, and no associations with HRQOL variables at the between-person level. One reason for this relatively minimal association with HRQOL metrics may be that the mean of the individual-specific average work hours reported (i.e., average of the average for each cluster), on workdays, was 4.82 hours (SD=2.23). Prior literature has found that working more than ten hours daily was associated with lower mental and physical health.”

*       P. 22: in their discussion of power limitations, the authors should also note the very small sample size of 92. Sample size at level 2 (in this case, number of persons) is also strongly related to power.

The discussion of statistical power limitations was updated to the below, in the first paragraph under the “limitations” section.

“Limitations regarding statistical power should be noted. Power to detect associations between the various activity types and HRQOL relevant metrics varied as a function of the number of times activities were reported. For activity types less frequently reported (such as “caring for others” and “socializing” in this sample), a larger number of individuals may need to be sampled to capture small yet potentially meaningful associations. Furthermore, our sample of n=92 and approximately 70 observations per person was sufficiently powered to detect small associations at the within-person level, but only moderately strong associations at the between-person level. Thus, small but possibly meaningful between-person associations between activity engagement frequency and HRQOL variables may have remained undetected. Finally, with only 92 individuals, specifying random slopes between each activity and a HRQOL variable at level one was infeasible. Our approach of using only random intercepts assumed that all individuals had the same associations between activity engagement and HRQOL. Future studies with larger sample sizes are needed to examine if our results replicate when using more complex models with random slopes, which allow for different associations between activity and HRQOL for each individual.”

*       The authors repeatedly discuss their findings in terms of "health promotion efforts," but after a full reading it is still not clear to me what the authors are referring to with this term. Providing more detail about how they envision these findings being used in practice may be helpful for driving home the importance of this study.
 Thank you for this feedback. In addition to the paragraph added to the introduction, the below text was added to the discussion under the “Health promotion implications” section.

“Collectively, study results provide a quantitative representation of the lived experience of T1D that could inform the clinical reasoning of practitioners of the Lifestyle Redesign® (LR) approach. For instance, average BG was found to be associated with frequencies of both napping and caregiving. With this in mind, for clients frequently engaged in napping or caregiving, LR practitioners may consider evaluating whether and how these activities relate to their diabetes management. If a client struggles to manage his/her diabetes in part because of responsibilities from caregiving, then part of the intervention may entail brainstorming strategies to carry out both duties so that they conflict less with one another. As another example, at the momentary level, “relaxing/chilling” and “fun/play/leisure” were typically associated with the lowest activity importance ratings. If a client is not engaging in optimal diabetes management because he/she is burned out from the collective load from responsibilities such as health management, part of an intervention may entail reinforcing the importance of relaxation/leisure, and/or brainstorming forms of relaxation/leisure aligned with the client’s values.”

EDITORIAL COMMENTS:
*       In general, I suggest sharpening up the writing throughout the introduction and discussion. Some specific issues are noted below.
*       P. 2: in the last sentence of the 2nd paragraph, I found that the comma placement made the sentence a bit difficult to follow. Please re-work this sentence.

The sentence was reworked to the below.

“Note that all these studies investigated the relationship between people’s average activity engagement across time and HRQOL.”

*       P. 3: I have a similar comment about comma placement for the first sentence of the 2nd paragraph on this page. Please re-work this sentence.

The sentence was reworked to the below.

“Investigation of the associations between engagement in daily activities and HRQOL relevant measures, at both the between-person and within-person levels, may help provide a more comprehensive picture of their relationship.”

*       P. 3: The first sentence of the 3rd paragraph uses "potential" multiple times. Please make more concise.

The sentence was reworked to the below.

“The greater clarity afforded by examining both within and between-person relationships between activity engagement and other HRQOL relevant variables can potentially yield insights for HRQOL interventions.”
